# Influence of leaf damage by the horse chestnut leafminer (*Cameraria ohridella* Deschka & Dimić) on mycorrhiza of *Aesculus hippocastanum* L.

**DOI:** 10.1007/s00572-018-0862-8

**Published:** 2018-08-25

**Authors:** J. Tyburska-Woś, K. Nowak, B. Kieliszewska-Rokicka

**Affiliations:** 1 Department of Mycology and Mycorrhiza, Kazimierz Wielki University, Al. Ossolinskich 12, 59-093 Bydgoszcz, Poland; 2 Institute of Dendrology, Polish Academy of Sciences, Parkowa 5, 62-035 Kórnik, Poland

**Keywords:** Aboveground herbivory, Mature trees, Arbuscular mycorrhiza, Soil dehydrogenase

## Abstract

In many parts of Europe, the white horse chestnut (*Aesculus hippocastanum* L.) has been attacked by the horse chestnut leafminer (*Cameraria ohridella* Deschka & Dimić), which causes premature leaf dieback. *A. hippocastanum* L. establishes mutualistic symbiosis with arbuscular mycorrhizal (AM) fungi. This study involved a comparison of mature *A. hippocastanum* individuals susceptible to *C. ohridella* and individuals resistant to this insect after a one-time treatment with a chemical preparation injected into the tree trunks 7 years before the investigation began. Concentration of macronutrients in soil and the activity of soil nonspecific dehydrogenase did not differ between soils under canopies of the treated and untreated trees. Concentrations of C and N were significantly higher in leaves of the treated than those of the untreated trees. The infestation by *C. ohridella* and defoliation of leaves of the untreated trees did not significantly influence the frequency and intensity of AM colonization compared to the chemically treated trees, although a tendency towards higher average AM colonization of roots of the untreated trees, infested by the herbivores, than roots of the non-infested trees was observed. The results also indicated a tendency for higher biomass of fine roots per soil volume under the trees treated against *C. ohridella* than under the trees invaded by the insect.

**Electronic supplementary material:**

The online version of this article (10.1007/s00572-018-0862-8) contains supplementary material, which is available to authorized users.

## Introduction

In the last decades, species of the genus *Aesculus* L. (Hippocastanaceae) in many parts of Europe have suffered attacks by the leaf-mining moth *Cameraria ohridella* Deschka & Dimić (Lepidoptera). The leaf parenchyma between the two epidermis layers is eaten by the insect larvae, and the infected leaves wither and fall prematurely (Takos et al. 2008; Thalmann et al. 2003). *Aesculus hippocastanum* is the main host of *C. ohridella* and is susceptible to the insect (Paterska et al. 2017). Methods of reducing the *C. ohridella* population include raking and destruction of chestnut leaves where the insect pupae hibernate, pheromone traps, and injections of insecticides into the tree trunk (Ferracini and Alma 2008).

Roots of most plant species are colonized by specialized soil fungi forming mycorrhizal symbiotic associations. *A. hippocastanum* is included among species forming symbiotic relationships with arbuscular mycorrhizal (AM) fungi (Glomeromycota) (Harley and Harley 1987; Wang and Qiu 2006); however, there is only limited information on how these mycorrhizas interact with biotic and abiotic factors. The few studies conducted so far on mycorrhizas of *A. hippocastanum* grown in urban and rural sites (Bainard et al. 2011; Tyburska et al. 2013; Karliński et al. 2014) showed generally lower-average AM colonization in urban than in rural environments, but the differences between the environments have been mostly not significant. Mycorrhizal fungi are dependent on carbon assimilated by the plant (Garbaye 1991); therefore, any factor that reduces the photosynthetic activity and allocation of carbon belowground can negatively influence mycorrhizal fungi (Andersen and Rygiewicz 1991; Cullings et al. 2005).

The results of several studies cited in review articles (e.g., Barto and Rillig 2010; Gehring and Bennett 2009; Gehring and Whitham 2002) indicated that defoliation or shoot decline can reduce mycorrhizal colonization of plant roots (in the majority of cases), increase mycorrhizal abundance (in a few cases), or have no influence. To date, the responses of arbuscular mycorrhizas of herbaceous plants, particularly grasses, to the influence of decline of aboveground parts have been studied most frequently. Among tree species investigated to date, research has focused on species that engage in ectomycorrhizal (ECM) symbiosis or dual mycorrhizal associations (ectomycorrhiza and arbuscular mycorrhiza) in the same root system, and diverse effects of defoliation have been reported, e.g., negative effect on ectomycorrhiza of *Castanea sativa* (Blom et al. 2009) and mountain birch (Saravesi et al. 2015), no significant influence on ectomycorrhiza of *Castanea dentata* and a hybrid *C. dentata* × *Castanea mollissima* (Rieske et al. 2003) or on arbuscular mycorrhiza of *Populus nigra* clones (Zampieri et al. 2016), and various influences on ectomycorrhiza and arbuscular mycorrhiza of different *Populus* species (Gehring and Whitham 2002). The authors of those studies suggested that the interactions between plants, herbivores, and mycorrhizal fungi can be influenced by plant and fungal species and by environmental factors.

The present study was performed to examine the relationship between leaf damage to mature *A. hippocastanum* trees (forming exclusively AM symbiosis) caused by *C. ohridella* and mycorrhizal colonization of their roots. To our knowledge, there has been no study yet on the interactions between *A. hippocastanum*, *C. ohridella* invasion, and AM colonization. We hypothesized that leaf damage can reduce the degree of colonization of tree roots by AM fungi. To explore this relationship, we compared AM colonization of the roots of mature trees attacked for several years by *C. ohridella*, the leaves of which are strongly damaged in mid-summer, to the AM colonization of roots of treated trees, with leaves that remained healthy until late autumn when they naturally fall. The presence of these two groups of horse chestnuts grown at the same site, under the same environmental conditions, provided a unique opportunity to identify the effect of the herbivore on mycorrhizas. Additionally, under the tree canopies, we examined the activity of soil nonspecific dehydrogenase (DHA), a cofactor-requiring enzyme that is almost exclusively intracellular and active only inside intact, living cells (Rossel et al. 1997). The enzyme activity is positively correlated with microbial biomass C, organic matter content, and basal respiration of soil (Garcia et al. 1997; Rossel et al. 1997). We used the DHA analysis to assess the overall vitality of the soil microbial community including AM fungi.

## Materials and methods

### Site description

The research was conducted in the Kórnik Arboretum, situated in west–central Poland (52° 14′ 30″ N, 17° 05′ 44″ E), which covers an area of 38 ha and represents one of the oldest and richest collections of trees and shrubs in Europe. We examined 25-year-old chestnut trees growing on two sides of a narrow alley in the southern part of the arboretum. The chestnut trees were planted in 1987 and, since 1999, have suffered because of attacks of *C. ohridella* such that their leaves withered and fell each year, usually from the beginning of July. In 2005, the trees growing on one side of the alley were treated with a chemical preparation containing 12% insecticide imidacloprid (*w*/*v*) and 8% fungicide tebuconazole as a one-time trunk injection therapy: “Gel to control *Cameraria ohridella* and *Guignardia aesculi* on horse chestnut trees using a microinjection technique” (Best-Pest, Jaworzno, Poland). The details of the treatment are described elsewhere (Jagiełło et al. 2018). The trees growing on the opposite side of the alley were not treated. The effect of the insecticide was long-lasting, so that 7 years after treatment, only a few leaves of the treated trees had visible traces of the insect feeding. The untreated trees had been attacked heavily by larvae of *C. ohridella*, and 100% of their leaves were infested by the middle of the summer (Supplementary, Fig. 1). The fungicide tebuconazole is considered not harmful for AM fungi, when used in modest amounts (e.g., Burrows and Ahmed 2007).

### Sampling

Soil and root samples for mycorrhizal evaluation and dehydrogenase assay were taken twice in 2012, on May 5, when the leaves of white horse chestnut trees were fully developed and no visible symptoms of the *C. ohridella* invasion were observed, and on October 5, when the leaves of the untreated trees were seriously damaged by the insects (Supplementary, Fig. 1) and the treated trees still had green, healthy leaves. Root samples of size about 15 × 15 cm to a depth of 10 cm were taken with a blade in three sampling sites under each of four treated trees and four untreated trees, about 70–100 cm away from tree trunk. Soil samples were used for soil pH, moisture, and dehydrogenase analyses, and roots were isolated for mycorrhizal assessment. The roots were traced from the trunk to be sure that the sampled roots had been connected with a given tree. Root pieces together with their derived, last-order roots were carefully removed, placed in plastic bags, and stored at − 18 °C until analysis. Soil-root samples for chemical analysis of soil and root biomass measurements were taken in 2018 on June 5, under treated and untreated trees, using a cylindrical sampler (diameter 5 cm, height 10 cm). Leaf samples were collected from the upper portions of tree canopies using a secateur on a long pole. Each sample consists of six leaves taken both near and away from the alley. Insect-damaged leaflets were not included. The leaves of each tree were pooled, dried at 65 °C, and powdered using a grinder.

### AMF colonization assessment

Roots were carefully washed, the fine roots were detached from coarse roots and cut into 1-cm segments, and randomly selected subsamples of 0.5 g each were stained according to the modified method of Kormanik and McGraw (1982). The roots were cleared in 10% KOH at 95 °C for 1.5–2.0 h, bleached for 1 h in alkaline hydrogen peroxide at room temperature, and stained with 0.05% trypan blue in lactoglycerol at 90 °C for 8 min. The stained roots were stored in a mixture of glycerol/lactic acid/water (1:1:1). Mycorrhizal colonization assessment was carried out using a Zeiss microscope at × 100–400 magnification according Trouvelot et al. (1986; http://www2.dijon.inra.fr/mychintec/Mycocalc-prg/download.html). At least 60 cm of fine roots for each subsample was analyzed to evaluate the frequency of mycorrhizal colonization of the root system (F%), the intensity of colonization of the root system (M%), the intensity of colonization in colonized root fragments (m%), the arbuscular abundance in the root system (A%), and the arbuscular abundance in colonized root fragments (a%).

### Root biomass measurement

Roots collected using the soil corer were washed under tap water, and the fine roots (< 2 mm diameter) were separated. The roots were dried at 70 °C for 48 h and weighted, and their density was expressed per 100 cm^3^ soil volume.

### Soil analyses

Soil was sieved to remove roots and was air-dried. The activity of nonspecific dehydrogenase was measured using a tetrazolium method developed by Von Thalmann (1968) and modified by Rossel et al. (1997). Samples of 2.5 g fresh weight were incubated in 5 ml of 0.5 Tris buffer (pH = 8.0), containing 1% of 2,3,5-triphenyltetrazolium chloride (TTC) as an electron acceptor, for 24 h at 30 °C in darkness. Two reference samples were analyzed to eliminate the influence of non-enzymatic absorbance: (1) soil + Tris buffer and (2) TTC + Tris buffer. Ethanol was used to extract the colored formazan from the incubation mixtures. The extract was measured spectrophotometrically at 480 nm. The soil samples for the determination of dry matter were dried at 120° for 24 h. Enzyme activity was expressed in nanomoles of 2,3,5-triphenyltetrazolium formazan/g dry soil/24 h. Soil pH was determined in soil-H_2_O and soil-0.1 M KCl suspension (2:2.5, *w*/*v*). The water content in soil was estimated using a standard operating procedure according to Hausenbuiller (1975). Concentrations of macronutrients in the soil were determined by a licensed laboratory. The Kjeldahl method was used to determine the nitrogen content with a Kjeldahl 2300, Tecator. Available phosphorus (P_2_O_5_) in soil was determined by a colorimetric method after extraction by the Egner-Riehm method (calcium lactate, pH = 3.5–3.7), and available potassium (K_2_O) and magnesium (Mg) were determined by flame emission mass spectrometry.

### Analysis of macroelements in leaves

Leaves were dried (65 °C, 48 h), ground in a ball mill, sieved to 1 mm particle size, mineralized (nitric acid digestion procedure) using a microwave mineralizer (Multiwave 3000; Anton Paar GmbH, Austria), and analyzed by a mass spectrometer (OptiMass 9500 ICP-TOF-MS; GBC) for phosphorus (P), potassium (K), calcium (Ca), and Mg concentrations. Additional tissue was analyzed by the 2400 Series II CHNS/O Elemental Analyzer (PerkinElmer, USA) for nitrogen (N) and C concentrations. The analysis was done in the Institute of Dendrology, Kórnik.

### Statistical analysis

Statistica version 9.0 (StatSoft, Inc.) was used for the statistical data processing. Two-way analysis of variance (ANOVA) was used to examine the effect of treatment (the trees treated with pesticides versus the untreated trees) and season (May 5 versus October 5) on mycorrhizal colonization of roots, pH, water content, and activity of nonspecific dehydrogenase in soil under the tree canopies, at a significance level of *p* < 0.05. In addition, we used one-way ANOVAs to evaluate the effects of the tree treatment on root biomass and concentrations of mineral elements in soil and leaves. Before analyses, the data were checked for normality (Shapiro-Wilk test) and homogeneity (Bartlett’s test), and the proportional data were transformed according to the Bliss formula (Snedecor and Cochran 1976): *x* = arcsin√(n% / 100) × 180 / *π*, where n% is the percent value. The table presents nontransformed data.

## Results

### Soil analyses

The pH of soil samples taken under canopies of all trees investigated was slightly alkaline, and no significant difference in soil pH was found among the soil samples taken under the pesticide-treated and untreated trees, both on May 5 and October 5 (${\mathrm{pH}}_{{\mathrm{H}}_2\mathrm{O}}$ = 7.6–7.7; pH_KCl_ = 7.1–7.3). The water content in soil samples also did not differ between the treated and untreated trees or between seasons. On May 5, it was 29.38% under the treated and 29.37% under the untreated trees, and on October 5, it was 30.44% under the treated trees and 30.38% under the untreated trees. One-way ANOVA did not show statistically significant differences in the concentrations of total N, available phosphorus (P_2_O_5_), potassium (K_2_O), and Mg in soil taken under canopies of both groups of trees (Table 1). Two-way ANOVA showed that soil DHA also did not differ significantly between the sampling sites (under the treated and untreated trees) and seasons. On May 5, the DHA was 0.47 μmol/g dry weight (dw) soil/24 h under the treated *A. hippocastanum* trees with healthy leaves and was 0.45 μmol/g dw soil/24 h under the untreated trees with damaged leaves. In October, the enzyme activity was 0.62 μmol/g dw soil/24 h under the treated trees and 0.60 μmol/g dw soil/24 h under the untreated trees.

**Table 1:** Concentration of macronutrients (total N and available forms of P, K, and Mg) in soil under canopies of *Aesculus hippocastanum* treated with insecticide and fungicide, and under untreated trees, and in leaves of the treated and untreated trees

	Treated	Untreated	One-way analysis of variance	
			*F*	*p*
Macronutrients in soil under *A. hippocastanum*				
P (P_2_O_5_) (mg/100 g)	18.63 (1.53)	21.43 (2.91)	2.16	0.21
K (K_2_O) (mg/100 g)	6.83 (1.04)	7.66 (0.28)	1.78	0.25
Mg (mg/100 g)	14.06 (3.49)	20.00 (2.00)	6.50	0.06
N (%)	0.25 (0.02)	0.29 (0.03)	3.35	0.14
Macronutrients in leaves of *A. hippocastanum*				
P (%)	0.19 (0.014)	0.16 (0.02)	3.55	0.13
Ca (%)	1.30 (0.07)	1.34 (0.15)	0.16	0.70
Mg (%)	0.28 (0.04)	0.29 (0.04)	0.02	0.88
K (%)	1.02 (0.08)	1.17 (0.27)	0.65	0.46
C (%)	48.55 (0.74)	46.83 (0.26)	14.4	0.01*
N (%)	2.51 (0.18)	1.92 (0.12)	22.51	0.00*
C/N	22.60 (2.00)	28.44 (1.77)	14.24	0.02*

Data are means ± SE (*n* = 3). For the concentration of macronutrients in soil, *df* effect = 1 and *df* error = 4; for the concentration of macronutrient in leaves, *df* effect = 1 and *df* error = 4

*Significant differences

### Leaf macronutrients

One-way ANOVA showed there were no significant differences in concentrations of P, Ca, Mg, and K in leaves of the treated and untreated trees (Table 1). Concentrations of carbon (C%) and nitrogen (N%) were significantly higher in leaves of the trees treated with pesticides and not infested by *C. ohridella* than in leaves of untreated trees, attacked by the insect (Table 1). The value of C/N was significantly higher in leaves of the untreated than in those of the treated trees (Table 1).

### Root biomass

The average biomass of fine roots (diameter < 2 mm) of *A. hippocastanum* trees that have been treated with chemical injection and were resistant to herbivory was higher than the average biomass of roots of the untreated trees (0.47 g dw/100 cm^3^ and 0.32 g dw/100 cm^3^, respectively), but the difference between the average values was not significant according to the one-way ANOVA.

### Mycorrhizal colonization

Roots of all the *A. hippocastanum* trees investigated were colonized by AM fungi that formed typical mycorrhizal structures inside the roots: vesicles, arbuscules, aseptate hyphae, and hyphal coils (Supplementary, Fig. 2). Both groups of trees revealed a high frequency (F%) of AM colonization (from 70.7% in roots of the untreated trees on May 5 to 86.8% in roots of the treated trees on October 5). Two-way ANOVA showed that differences in all the mycorrhizal indices (F%, M%, m%, A%, a%) did not differ statistically between the treated and untreated trees and between the sampling seasons (May 5, October 5) (Table 2). Nevertheless, there was a tendency for marginally significant (*p* = 0.06–0.07) higher average intensity of AM colonization (M% and m%) in roots of the untreated trees than in roots of the trees treated with pesticides (Table 2).

**Table 2:** Mycorrhizal colonization of roots of *Aesculus hippocastanum* L. trees annually defoliated because of infestation by *Cameraria ohridella* (untreated trees) and of trees that have been treated with one-time trunk injection chemical therapy (treated trees) in two sampling times (in 2012, on May 5 and October 5), and the interactions between the treatment and season on mycorrhizal colonization

Mycorrhizal colonization	Two-way analysis of variance			May 5, 2012		October 5, 2012	
	Factor	*F*	*p*	Treated	Untreated	Treated	Untreated
F%	Treatment	1.87	0.19	84.02	70.69	86.80	86.38
	Season	3.40	0.09	(13.29)	(15.72)	(12.66)	(13.29)
	Treatment × season	1.62	0.23				
M%	Treatment	4.41	0.06	1.68	4.03	2.29	4.08
	Season	0.36	0.56	(0.79)	(4.16)	(1.67)	(3.10)
	Treatment × season	0.05	0.83				
m%	Treatment	4.02	0.07	1.95	5.10	3.64	4.57
	Season	0.31	0.58	(0.76)	(4.64)	(1.86)	(3.22)
	Treatment × season	0.03	0.86				
A%	Treatment	0.37	0.55	0.01	0.01	0.01	0.03
	Season	2.80	0.12	(0.02)	(0.02)	(0.02)	(0.05)
	Treatment × season	0.72	0.41				
a%	Treatment	0.08	0.78	1.10	0.41	1.02	1.27
	Season	1.59	0.23	(2.66)	(0.81)	(1.29)	(2.00)
	Treatment × season	0.57	0.47				

F% = the frequency of mycorrhizal colonization of roots; M% = the intensity of mycorrhizal colonization in the root system; m% = the intensity of mycorrhizal colonization in colonized root fragments; A% = the arbuscular abundance in the root system; a% = the arbuscular abundance in colonized root fragments. Data are means ± SE (*n* = 4); *df* effect = 1; *df* error = 12

## Discussion

The results of this study showed that annual infestation and defoliation of mature white horse chestnut by the horse chestnut leafminer *C. ohridella* did not influence the frequency (F%) and intensity of mycorrhizal colonization (M%, m%) of the tree roots when compared to the roots of control trees that were not attacked by the insect. Nevertheless, a tendency towards a higher mycorrhizal intensity was observed in roots of infested than in those of non-infested trees (Table 2). Moreover, this study indicated that the trees infested by *C. ohridella* had significantly lower concentrations of C and N in leaves (Table 1) and tended to have a lower biomass of fine roots than the healthy trees, although not significantly so. Diverse responses to defoliation or grazing on mycorrhizas (negative, positive, or neutral) have been reported in the literature, but data on interactions between herbivory and mycorrhizas of deciduous trees are very scarce. The meta-analysis of Barto and Rillig (2010), based on data concerning numerous plant species, revealed that overall, herbivory reduced mycorrhizal colonization by about 3%, i.e., less than biologically meaningful amounts, while in a group of seven deciduous tree species (ectomycorrhiza and ectomycorrhiza/arbuscular mycorrhiza) included in the analysis, the average mycorrhizal colonization was reduced by 8%. A pronounced decrease of ECM abundance and richness (70–80%) caused by defoliation was reported by Saravesi et al. (2015) in roots of mountain birch in a forest in Finland. The authors suggested a marked reduction in the carbon flow from plants to soil fungi following defoliation.

To our knowledge, the present study is the first investigation on the response of arbuscular mycorrhiza of *A. hippocastanum* to defoliation caused by the insect *C. ohridella* and, probably, the first in terms of interactions between herbivory and arbuscular mycorrhiza in roots of a deciduous tree species forming exclusively arbuscular mycorrhiza. The long-term effective protection of the insecticide imidacloprid for the chestnut leaves of the *A. hippocastanum* grown in the Kórnik Arboretum against *C. ohridella* attacks is reported in detail by Jagiełło et al. (2018). An advantage of our investigation is the unique study site that provided the same soil properties for the *A. hippocastanum* trees studied. The soil pH, the soil moisture, and the concentration of macronutrients (N, P, K, Mg) did not differ under canopies of the treated and untreated trees, providing uniform edaphic conditions (Table 1). In root samples of the trees attacked by *C. ohridella* and the trees resistant to the insect, functional AM symbiosis was indicated by the presence of arbuscules, vesicles, intramatrical hyphae, and coils (Supplementary, Fig. 2) with high frequency of mycorrhizal colonization recorded in roots of both the groups of trees (70.7–86.8%) (Table 2). Arbuscules, the structures involved in the nutrient exchange between the fungus and the plant, were present in roots of all the trees investigated, and their abundance did not differ by tree group or season, although the abundance of arbuscules was relatively low on both of the study dates. Arbuscules are ephemeral structures persisting for only several days in root cells. The performance and activity of arbuscules of *A. hippocastanum* is strongly connected with tree phenology (unpublished data). In opposition to the results of Saravesi et al. (2015) who revealed a 70–80% decrease of ECM abundance of mountain birch, our study did not show any negative influence of herbivory and defoliation on AM colonization of *A. hippocastanum*. A slightly higher colonization intensity (M%, m%, Table 2) in roots of the trees attacked annually by the herbivore suggests a tolerance of AM fungal symbionts to changes in plant physiological status caused by *C. ohridella*. The lack of statistically significant differences in intensity of mycorrhizal colonization found in this study could be influenced by a limited number of trees (repetitions) available at the study site in the Kórnik Arboretum and high variability in the colonization level between particular root samples taken under an individual tree.

The significantly higher concentration of the growth-limiting macroelements C and N in leaves of the trees resistant to *C. ohridella* after pesticide treatment than in the untreated trees (Table 1) is in agreement with the tendency to a higher biomass of roots (diameter ≤ 2 mm) in the treated than in the untreated trees found in our study, and with the significantly greater increase in height, in diameter at breast height, and in basal area increment recorded for the same white horse chestnut trees recorded in 2014 by Jagiełło et al. (2018). Thus, the growth of the above- and belowground parts of the *A. hippocastanum* trees grown under the same environmental conditions seems to be tightly associated, in accordance with the theory of Pearsall (1927). Similarly, infestation of leaves of *A. hippocastanum* by *C. ohridella* was shown to reduce the photosynthetic activity and allocation of carbohydrates to roots (Takos et al. 2008; Percival et al. 2011) and to decrease the growth of infested trees (Percival et al. 2011) as well as to lower the quality and germination of seeds (Takos et al. 2008) as compared to non-infested trees. Reduced growth of shoots and roots after defoliation has been confirmed by laboratory experiments with seedlings of the ECM deciduous tree *Betula pubescens* (Markkola et al. 2004; Kytöviita 2005).

As the roots of the healthy *A. hippocastanum* trees in our study produced a higher biomass of fine roots than the infested trees, it might be possible that the development of mycorrhizal fungi cannot keep up with the development of roots of the healthy trees as was presented by Torti et al. (1997). Mycorrhizal fungi differ in metabolic activity and in nutritional requirements, so it also cannot be excluded that the trees producing lower amounts of C might be colonized by different fungi, with lower C requirements, than the healthy trees. Such a case was reported for mountain birch by Saravesi et al. (2015), who observed a shift in ECM fungal species in the tree rhizosphere after defoliation.

Carbohydrates released by roots to soil form an easily available source of energy which feeds soil microorganisms and stimulates microbial respiration (i.e., Högberg et al. 2001). Soil DHA has been shown to be significantly related to the concentration of nonstructural carbohydrates in fine roots of *Pinus sylvestris* grown under uniform environmental conditions (Kieliszewska-Rokicka et al. 2003). The present study indicated that tree defoliation by *C. ohridella* and season (May/October) did not significantly affect soil microorganisms’ vitality in the root zone of *A. hippocastanum* which suggests that fine roots of *A. hippocastanum*, whether infected by *C. ohridella* or healthy, deliver similar quantities of carbohydrates to soil. Kytöviita (2005) found comparable results, reporting that defoliation of seedlings of the ECM species *Betula pendula* did not decrease the carbon concentration of fine roots, which carbon can be transported to mycorrhizal fungi and released to soil. The three-way interactions between herbivory, plants, and mycorrhizal fungi are complicated by defensive responses of the organisms and by conditions of the environment.

## Electronic supplementary material

Supplementary Figure 1 (PDF 569 kb)

Supplementary Figure 2 (PDF 447 kb)
