# Supplementary material for: Influence of leaf damage by the horse chestnut leafminer (Cameraria ohridella Deschka & Dimić) on mycorrhiza of Aesculus hippocastanum L
Source: Mycorrhiza. 2018 Aug 25;29(1):61–7. doi: 10.1007/s00572-018-0862-8 (PMC6311180; doi:10.1007/s00572-018-0862-8)
Supplement: Supplementary file 1 — (PDF 569 kb) [file 572_2018_862_MOESM1_ESM.pdf]

Tyburska-Woś J., Nowak K., Kieliszewska-Rokicka B.

Influence of leaf damage by the horse chestnut leafminer (*Cameraria ohridella* Deschka & Dimić) on mycorrhiza of *Aesculus hippocastanum* L.

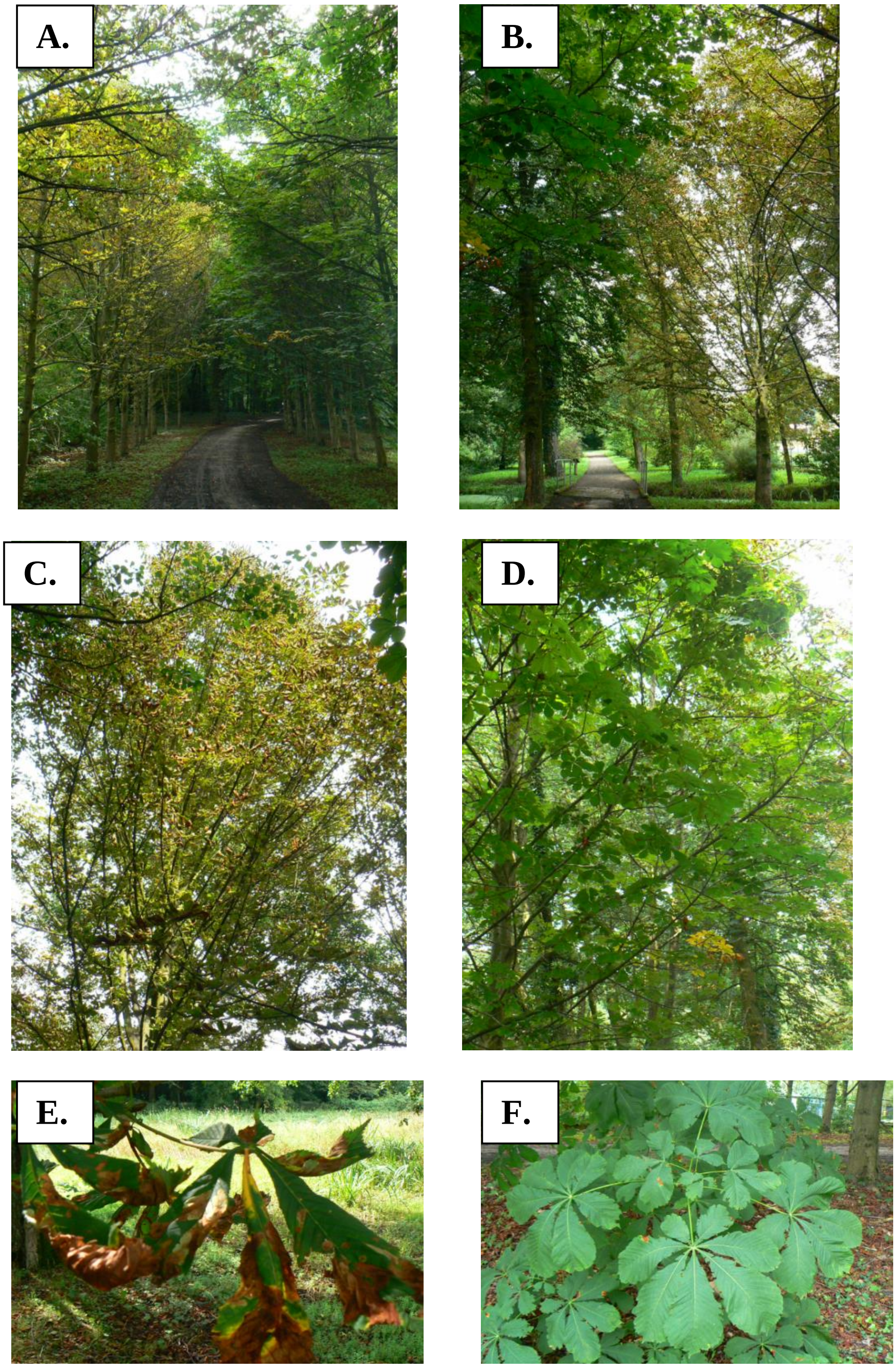

Supplementary Fig. 1. A,B – Alley of white horse chestnuts (*Aesculus hippocastanum* L.) in Kórnik Arboretum (view from two sides): trees treated with chemical preparation against *Cameraria ohridella* and *Guignardia aesculi* (green leaves) and not treated (damaged leaves); C, E – horse chestnut infested by C. ohridella; D, F – horse chestnut treated with chemical preparation.
