# Supplementary material for: Influence of leaf damage by the horse chestnut leafminer (Cameraria ohridella Deschka & Dimić) on mycorrhiza of Aesculus hippocastanum L
Source: Mycorrhiza. 2018 Aug 25;29(1):61–7. doi: 10.1007/s00572-018-0862-8 (PMC6311180; doi:10.1007/s00572-018-0862-8)
Supplement: Supplementary file 2 — (PDF 447 kb) [file 572_2018_862_MOESM2_ESM.pdf]

Tyburska-Woś J., Nowak K., Kieliszewska-Rokicka B.
Influence of leaf damage by the horse chestnut leafminer (*Cameraria ohridella* Deschka & Dimić) on mycorrhiza of *Aesculus hippocastanum* L.

ARBUSCES

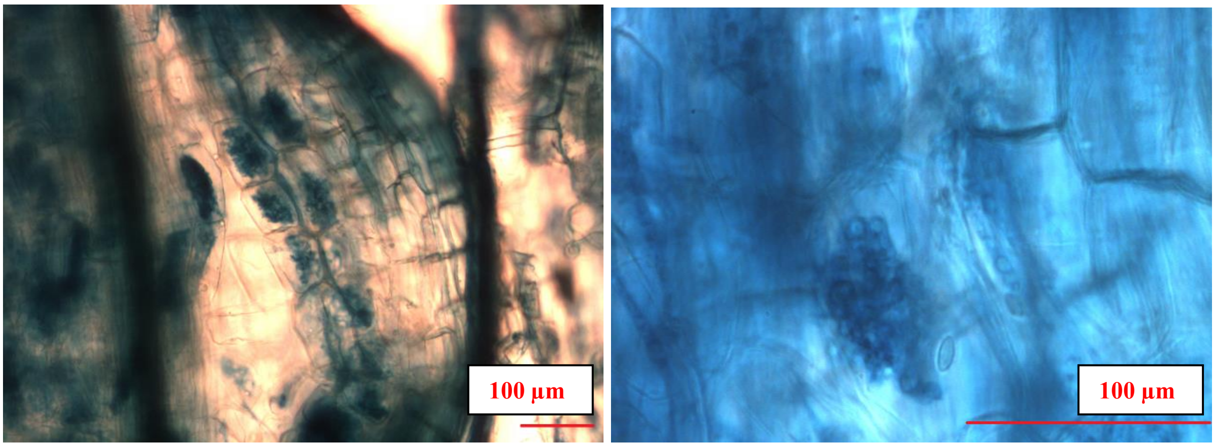

VESICLES

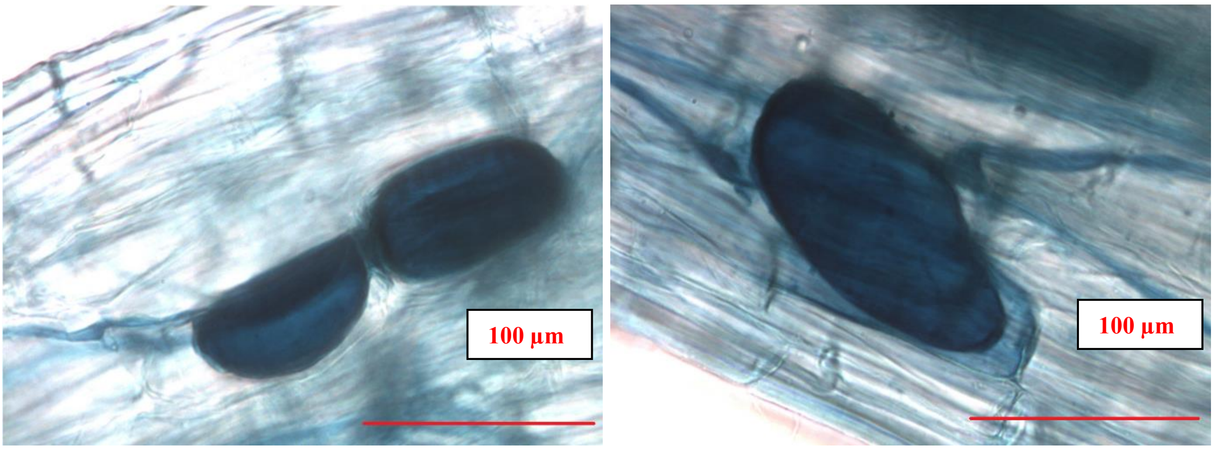

VESICLES

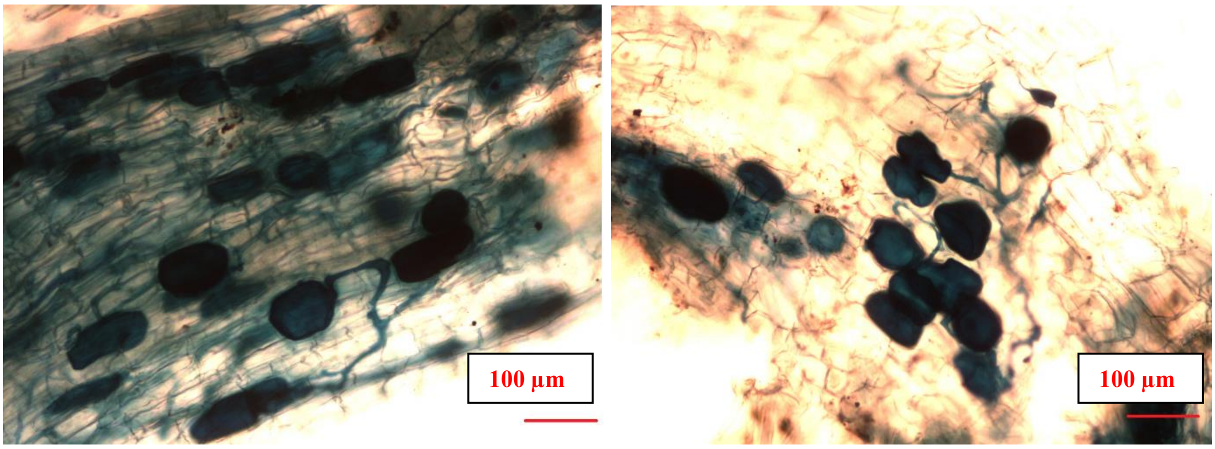

Supplementary, Fig.2. Arbuscular mycorrhizal colonization of roots of *Aesculus hippocastanum* L.
